# Mitochondrial ATP Biosynthesis Is Negatively Associated with FFA in Cardiac and Skeletal Muscle During the Development of Obesity in a Rodent Model

**DOI:** 10.3390/ijms26188768

**Published:** 2025-09-09

**Authors:** Vianey Nava-Aguilar, Angelica Ruiz-Ramirez, Zeltzin Alejandra Ceja-Galicia, Maria de la Luz Hernandez Esquivel, Magalena Cristobal Garcia, Roxana Carbó Zabala, Guillermo-Celestino Cardoso-Saldaña, Mohammed El-Hafidi

**Affiliations:** 1 Departamento de Biomedicina Cardiovascular, Ciudad de Mexico 14080, Mexico; viana_aguilar@yahoo.com.mx (V.N.-A.); angelica.ruiz@cardiologia.org.mx (A.R.-R.); alejandra.ceja@cardiologia.org.mx (Z.A.C.-G.); magdalena.cristobal@cardiologia.org.mx (M.C.G.);; 2 Departamento de Bioquímica, Ciudad de Mexico 14080, Mexico; maria.esquivel@cardiologia.org.mx; 3 Departamento de Endocrinologia, Instituto Nacional de Cardiología Ignacio Chávez, Juan Badiano 1, Seccion XVI, Tlalpan, Ciudad de Mexico 14080, Mexico

**Keywords:** ATP synthesis rate, free fatty acid, mitochondria, obesity, sucrose diet

## Abstract

Many factors related to obesity can impact how mitochondria produce ATP, such as the uncoupling of oxidative phosphorylation (OXPHOS) caused by proton leaks from built-up free fatty acids (FFA), the increased levels of uncoupling proteins (UCPs), and changes in the levels of ATPase inhibitory protein factors 1 (IF1). Therefore, the present study aimed to assess the rate of ATP synthesis in mitochondria isolated from skeletal and cardiac muscle from animal models of sucrose diet-induced obesity at different time periods. Short periods of sucrose intake (6 and 12 weeks) are sufficient to induce fat accumulation, hypertriglyceridemia, and high plasma FFA. However, a significant decline in the ATP synthesis rate starts to be obvious in mitochondria from skeletal muscle after 24 weeks of sucrose consumption. This impairment of ATP synthesis is associated with increased FFA in skeletal muscle homogenate. ATP synthesis rates in both skeletal and cardiac muscle were found to be sensitive to oleic acid and GDP, a physiological inhibitor of UCPs that has been shown to increase with aging. In addition, a sucrose diet increases the IF1 content in both skeletal and heart muscle, probably to avoid the hydrolytic activity of ATP synthase. In mitochondria from heart muscle, a decrease in the ATP synthesis rate was only observed according to the age in both groups of rats, and it was not affected by sucrose feeding. Our results suggest that the decline of the ATP synthesis rate in mitochondria from skeletal muscle can be due to the accumulation of FFA in skeletal muscle tissue as uncouplers, and the IF1 overexpression induced by the sucrose diet is a response mechanism to avoid the ATP hydrolysis and to save the energy charge reduced by FFA-uncoupling OXPHOS.

## 1. Introduction

Fatty acids and glucose, through fatty acid beta-oxidation and glycolysis, respectively, are the main nutrients and fuels that drive mitochondrial ATP generation in both skeletal and cardiac muscle [1]. In mitochondria, ATP biosynthesis is further regulated by ATP demand, by substrate supply, and by the coupling of oxidative phosphorylation (OXPHOS) to the proton gradient resulting from mitochondrial electron transfer. However, it has been described that excess FFAs due to an obesogenic diet characterized by high caloric intake, particularly fat and sucrose, uncouple OXPHOS through multiple mechanisms, including activation of uncoupling proteins (UCPs), modulation of the ATP/ADP antiporter, and increased ROS generation [2]. UCPs have been described to be involved in a dual effect on cell function: On the one hand, they protect against lipotoxicity, and on the other hand, they exhibit an uncoupling effect on the biosynthesis of ATP [3]. UCP2 and UCP3, specific uncoupling proteins of muscles, have been described to mediate energy expenditure via uncoupling, thereby dissipating the mitochondrial proton gradient necessary for ATP synthesis [4]. In addition, UCP3 overexpression is considered protective against lipotoxicity [5]. In skeletal muscle, UCP3 has been found to be up-regulated when the mitochondria exceed their capacity to oxidize fatty acids and down-regulated when oxidative capacity is high or improved, suggesting its role in fatty acid oxidation [6,7]. In heart muscle from high-fat-fed mice, UCP3 has been found to contribute to energy deficiency, and it is associated with heart failure, probably by FFA-induced OXPHOS uncoupling [8,9].

The increased supply of FFA by high nutrient availability from an obesogenic diet significantly contributes to lipid accumulation and subsequent mitochondrial and cellular dysfunction in both skeletal and heart muscle [10,11]. A detailed review on increased intramuscular lipid content and its association with mitochondrial dysfunction has recently been published [12]. In contrast, nutrient restriction maintains mitochondrial function as a major site of ATP biosynthesis in both skeletal and heart muscles [13]. The FFA accumulation in both skeletal and heart muscle tissues may depend on the type and the duration of the obesogenic diet. The impact of an obesogenic diet on FFA has been extensively studied, but few studies have focused on the short- and long-term effects on ATP biosynthesis in skeletal and heart muscles. These studies reveal that short-term exposure to high-fat diets (HFDs) or obesogenic diets has been shown to induce rapid changes in FFA profiles and metabolic parameters. Long-term exposure to obesogenic diets leads to more significant and enduring alterations in FFA metabolism and lipid profiles [14]. The duration of the diet-induced obesity may play a crucial role in the degree of metabolic syndrome disorders by disrupting energy metabolism through mitochondrial dysfunction, determining the extent of impairment in ATP biosynthesis [15]. During the development of obesity and metabolic syndrome, tissue FFA may increase chronically and may affect mitochondrial function in both skeletal and heart muscle. Moreover, the very long period of obesogenic diet involves aging, which may play a critical role in the exacerbation of metabolic diseases with the disruption of energy metabolism through mitochondrial dysfunction [16]. The prolonged intake of a sucrose diet (20 to 24 weeks) has been described to induce FFA accumulation in the liver, which is related to obesity and oxidative stress without mitochondrial dysfunction [17,18]. Whereas short-term sucrose intake may not lead to significant changes in mitochondrial function, it can accelerate the process of aging, as suggested elsewhere [16,19]. Moreover, it remains to establish the effect of short-, long-, and very-long-term sucrose intake on the decline of ATP biosynthesis in skeletal and heart muscle. Therefore, the present study aims to clarify the order in which changes in FFA content may affect the mitochondrial ATP biosynthesis in skeletal and heart muscle during the development of obesity induced by a high-sucrose diet in Wistar rats. We hypothesized that mitochondrial ATP synthesis would show a negative correlation with FFA accumulation in both tissues.

## 2. Results

### 2.1. General Characteristics of Animals

Table 1 shows that the administration of sucrose at 30% in the drinking water of Wistar rats increases intra-abdominal fat accumulation and plasma triglyceride and FFA concentrations at the end of different sucrose treatment periods in the animals. It also shows that all measured variables tend to increase with age, but not all reached statistically significant difference. Adipose tissue amount starts to display a significant difference (*p* < 0.01) between control and SF rats after the sucrose treatment period of 12 weeks. Triglycerides, on the other hand, start to increase in a short period of 6 weeks of sucrose consumption in plasma from SF rats compared with C rats, and this increase in triglycerides becomes more significant during the two long periods of sucrose treatment (24 and 56 weeks). In regard to plasma FFA, Table 1 shows that their concentrations in plasma start to significantly increase at 12 weeks.

We found that the more sucrose the animals consumed, the greater the difference in TG, FFA, and fat accumulation between the two groups. In regard to the body weight, Table 1 does not show significant changes between the two groups during the first 24-week period of sucrose diet. However, after 56 weeks of sucrose treatment, the body weight of SF rats increased significantly compared to that of the corresponding C rats. With regard to plasma glucose and cholesterol concentrations, no differences were observed between animals, and the treatment period had no influence on these two parameters. (Table 1).

### 2.2. Skeletal and Heart Muscle FFA Content

**Figure 1:**
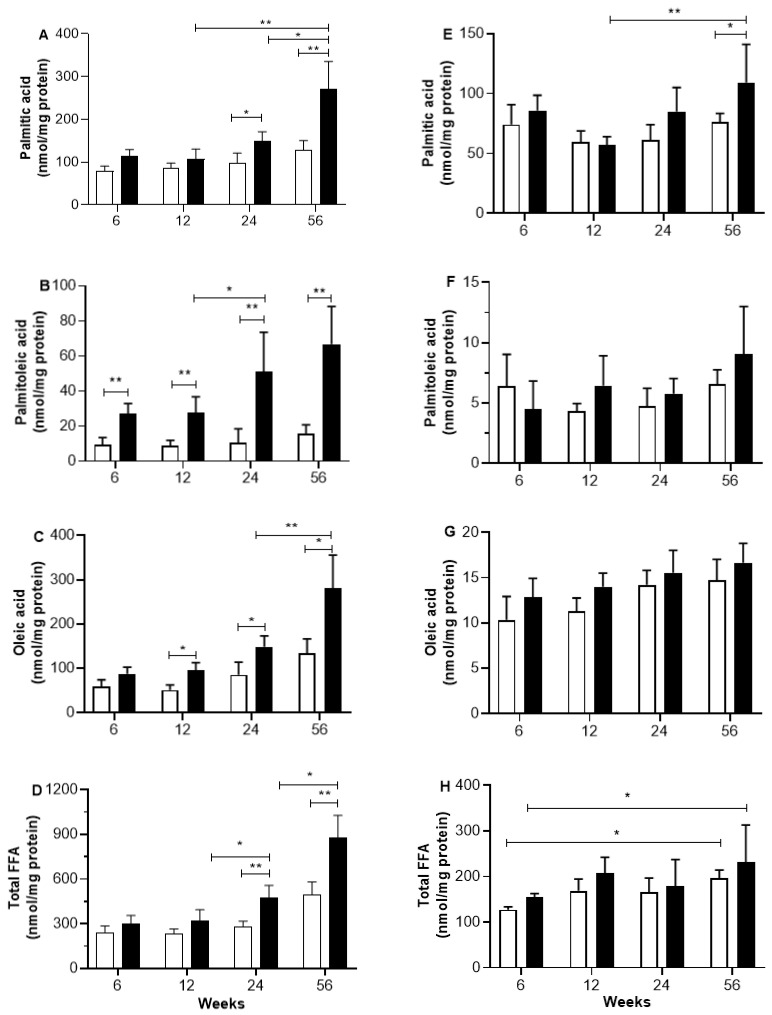
This chart illustrates the impact of varying sucrose intake periods on the levels of FFA in the homogenate of skeletal (**A**–**D**) and heart muscle (**E**–**H**). Black bars correspond to SF rats, and open bars correspond to C rats. For more details of FFA extraction and their GC analysis, see Section 4. The values correspond to the mean ± SD of 6–7 different samples from different animals. Horizontal square brackets indicate the significant differences between groups and the corresponding *p*-value (* *p* < 0.05 and ** *p* < 0.01).

Figure 1 shows the FFA composition in skeletal and heart muscle homogenates induced by different periods of sucrose diet. Palmitic, palmitoleic, and oleic acid levels start to rise noticeably in the skeletal muscle of rats after 12 weeks on a sucrose diet, and the gap between C and SF rats grows larger as the sucrose diet continues. In C rats, on the other hand, there is an age-related increase in the concentrations of the three fatty acids mentioned above, without reaching a statistically significant difference. Finally, the changes in the amounts of each fatty acid we found are shown in the rise in total FFA levels, which are linked to both the length of the sucrose diet and the age of the rats, as seen in Figure 1D. In heart muscle samples, the levels of palmitic, palmitoleic, and oleic acids did not change with age or during the sucrose treatment period. Similarly, there were no changes in the total FFA levels in heart muscles at any time during the sucrose diet. In the same way, no changes in the concentration of total FFA were found in heart muscles during any period of sucrose diet (Figure 1H).

### 2.3. Mitochondria Oxygen Consumption Rate (OCR)

Table 2 shows the OCR of mitochondria oxidizing succinate as a substrate of complex II in both states III and IV and in the presence of rotenone. In skeletal muscle mitochondria, the OCR oxidizing succinate only and in the presence of ADP (state III) decreases with age in C rats and also decreases as the duration of sucrose intake in SF rats increases. At 56 weeks of age, OCR of mitochondria from skeletal muscle of C rats is significantly reduced compared to 6 weeks of age (69.9 ± 10.8 vs. 121.0 ± 7.2 nmol O_2_/min/mg protein; *p* < 0.05). In SF rats after 56 weeks of sucrose intake, mitochondrial OCR is also significantly reduced compared to 6 weeks of sucrose intake (50.08 ± 7.1 vs. 115.6 ± 9.15 nmol O_2_/min/mg protein; *p* < 0.01).

On the other hand, in state IV after ADP depletion, mitochondrial OCR increased significantly after 56 weeks of sucrose intake compared with 6 weeks of sucrose intake (27.4 ± 2.8 vs. 22.1 ± 3.1 nmol (O_2_)/min/mg protein; *p* < 0.05). Whereas in skeletal muscle from C rats, the aging did not affect state IV mitochondrial OCR.

In heart muscle mitochondria, the OCR oxidizing succinate in the presence of ADP (state III) decreased with age in C rats (61.7 ± 8.9 vs. 118.8 ± 10.2 nmol (O_2_)/min/mg protein; *p* < 0.05) and with the duration of sucrose intake in SFR (50.8 ± 16.4 vs. 95.6 ± 14.8, *p* < 0.05). However, no differences in OCR were found between C and SF rats at any age or sucrose intake period. When looking at state IV, which happens when ADP is used up, there were no differences in OCR between mitochondria from C and SF rats at any age or during any period of sucrose intake.

### 2.4. ATP Biosynthesis in Skeletal and Heart Muscles

**Figure 2:**
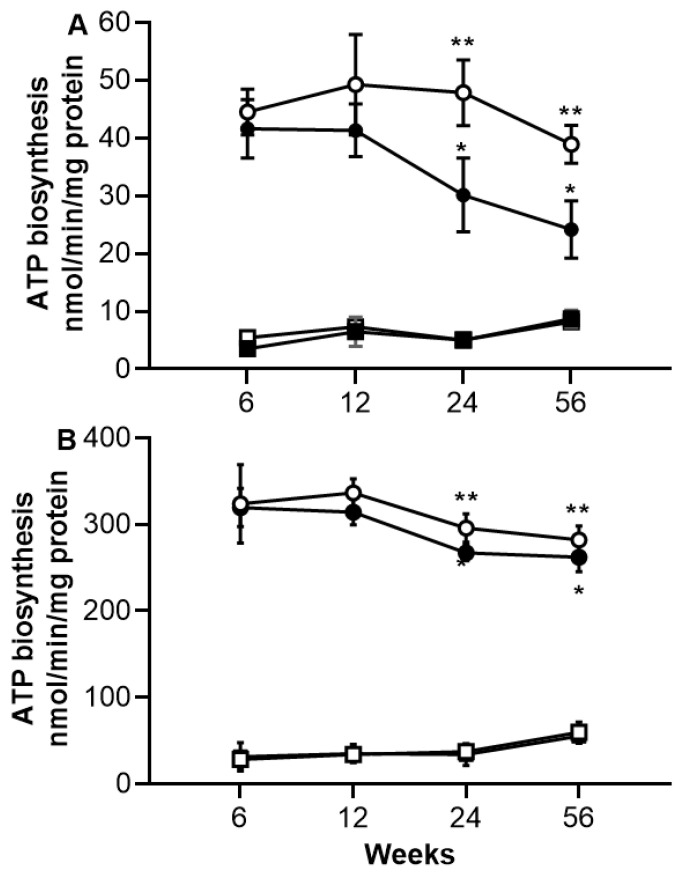
ATP biosynthesis at different periods of sucrose consumption and age in mitochondria from skeletal muscle (**A**) and heart muscle (**B**). Black circles correspond to SF rats, and open circles correspond to C rats. The black square corresponds to oligomycin-sensitive ATP biosynthesis of SF rats, and the open square corresponds to oligomycin-sensitive ATP biosynthesis of C rats. For more information about the experiment, see Section 4. The values correspond to the mean ± SD of 8 different samples from different animals. ** *p* < 0.01 from SF rats, and * *p* < 0.05 corresponds to the difference between ages or periods of sucrose diet.

In the skeletal muscle, the short periods of sucrose diet (6 and 12 weeks) did not influence the ATP synthesis rate (Figure 2A). However, long periods of an unhealthy diet (24 and 56 weeks) greatly reduced the ATP production in the mitochondria of skeletal muscle from SF rats compared to C rats, with rates of 30.1 ± 6.4 vs. 47.8 ± 5.6 nmol/min/mg protein at 24 weeks (*p* < 0.001) and 24.2 ± 4.9 vs. 38.8 ± 3.29 nmol/min/mg protein at 56 weeks (*p* < 0.001). In addition, a slight decrease in the ATP synthesis rate was observed according to the age in C rats but did not reach a significant difference. This ATP synthesis rate in mitochondria from skeletal muscle is sensitive to oligomycin independently of the period of sucrose treatment or of the age in C rats (Figure 2A).

In the heart muscle, the ATP synthesis rate was examined, and Figure 2B shows that there is no difference in ATP synthesis rate between mitochondria from C and SF rats at the end of any sucrose treatment period. However, a slow decrease in ATP synthesis rate is observed in C rats according to age without reaching a significant difference. As described above for skeletal muscle, the ATP synthesis rate in heart muscle was found to be sensitive to oligomycin, and this sensitivity is independent of the period of the diet rich in sucrose or of the age of the animals (Figure 2B).

### 2.5. Effect of Oleic Acid on ATP Synthesis Rate in Skeletal and Heart Muscles

**Figure 3:**
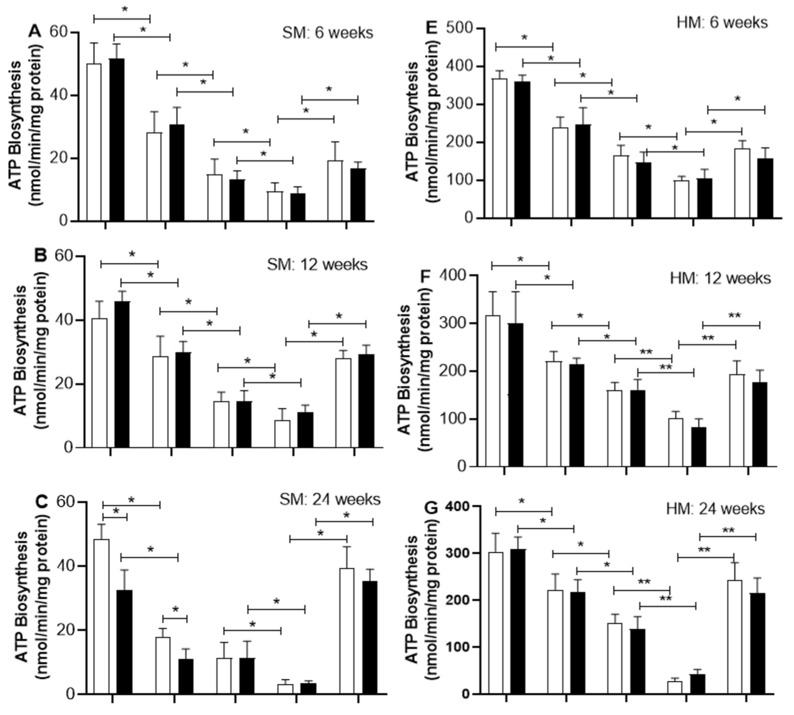

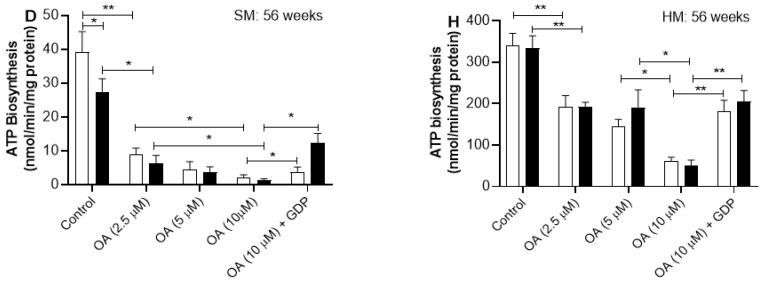
Effect of oleic acid on ATP synthesis rate in mitochondria from C rats (open bar) and from SF rats (black bars). **A**–**D** correspond to skeletal muscle (SM). **E**–**H** correspond to heart muscle (HM). The effect of oleate on ATP production was tested in mitochondria from skeletal muscles (SM) and heart muscle (HM) of rats that had been treated with sucrose for 6, 12, 24, and 56 weeks, compared to normal rats. GDP was assayed to inhibit UCPs at 0.5 mM in the presence of 10 μM oleic acid. Data are means ± SD of 6 to 7 experiments performed in separate mitochondria preparations. Horizontal square brackets indicate the significant differences between groups and the corresponding *p*-value: * *p* < 0.01 and ** *p* < 0.001.

Figure 3 shows that oleic acid inhibits the biosynthesis of ATP in a dose-dependent manner in mitochondria from skeletal and heart muscles. After a short time on a sucrose diet (6 and 12 weeks), oleic acid, at all tested amounts, reduces the ATP production rate in the mitochondria of skeletal muscle in C rats just like it does in SF rats (Figure 3A,B). However, after a long period of sucrose diet (24 and 56 weeks), the ATP synthesis in mitochondria from skeletal muscle of both C and SF rats shows more sensitivity to oleic acid at any concentration assayed than that from mitochondria of 6- and 12-week-period treatment (Figure 3C,D). At long and very long periods of sucrose treatment (24 and 56 weeks), oleic acid at high concentration (10 µM) exhibits an inhibition of approximately more than 90% of the ATP synthesis rate in mitochondria from skeletal muscle of both C and SF rats (Figure 3C,D).

At 10 µM, oleic acid inhibition of ATP biosynthesis in mitochondria from skeletal muscle is abolished by 0.5 mM of GDP, a physiological inhibitor of UCPs at all periods of sucrose consumption being more appreciated in SF than C rats at the end of 56 weeks of sucrose diet and age.

In heart muscle, oleic acid slows down the rate of ATP production in a way that depends on the amount used, and it affects both C and SF mitochondria the same (Figure 3). At a short period of sucrose treatment, oleic acid at high concentration (10 µM) partially inhibits the ATP synthesis (Figure 3E,F). Whereas at a long period of sucrose diet (24 and 56 weeks), 10 µM oleic acid significantly inhibits the biosynthesis of ATP by approximately 90% in both SF and C rats. The inhibition of the ATP biosynthesis by oleic acid at 10 µM was partially abolished by GDP at a short period of sucrose diet (6 and 12 weeks). Whereas after a long period of sucrose diet, the ATP synthesis rate is recuperated by approximately 90% in both types of mitochondria (Figure 3H,G).

### 2.6. The Analysis Focuses on Proteins Involved in ATP Biosynthesis

**Figure 4:**
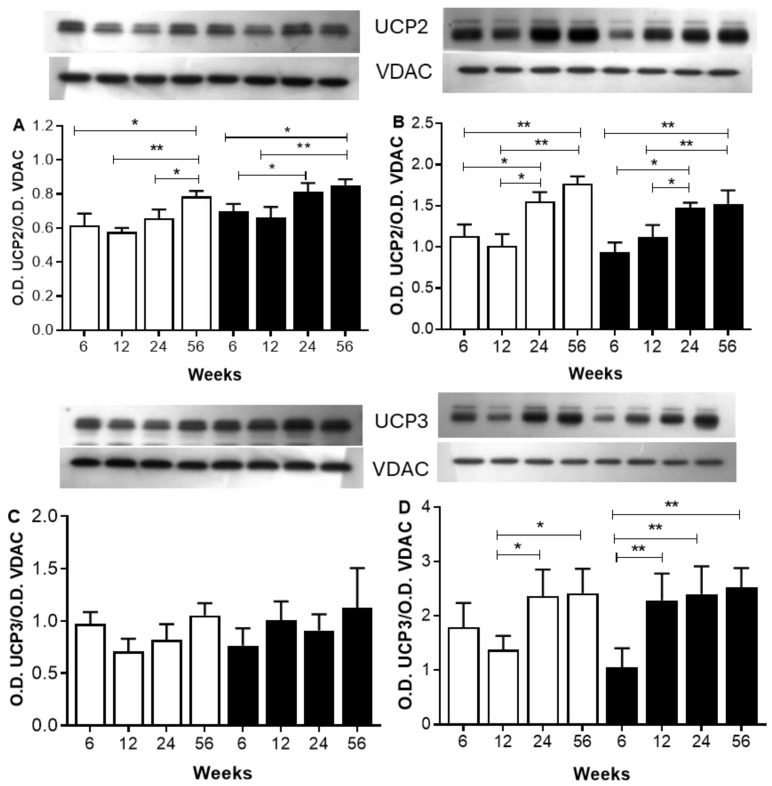
Western blot analysis of UCP2 and UCP3 in mitochondria from skeletal (**A**,**C**) and heart muscle (**B**,**D**). The open bars correspond to mitochondria from C rats and the black bars correspond to mitochondria from SF rats. Forty µg of proteins were electrophoresed on SDS-PAGE with 12% acrylamide, electroblotted, and incubated with anti-UCP2 and UCP3 antibodies. The optical density (O.D.) of the UCPs was normalized to the O.D. of VDAC as the leading control in different mitochondria. The values correspond to the ratio of the optical density (O.D.) of the UCPs to the O.D. of VDAC. Each value is the mean ± SD of 4 independent mitochondria preparations of each animal. Horizontal square brackets indicate the significant differences between groups and the corresponding *p*-value. * *p* < 0.05, ** *p* < 0.01.

Figure 4 shows a Western blot analysis of UCP2 and UCP3 in skeletal and heart muscles after different periods of sucrose consumption compared to control animals. In the mitochondria of skeletal muscle, looking at UCP2 and UCP3 showed no important differences in their levels between the C and SF rat groups, regardless of age or diet (Figure 4A). In mitochondria from the hearts of C rats, the O.D. of UCP2 and 3 bands increases as well as the age. In mitochondria from heart muscle of SF rats, the O.D. of UCP2 and three increases as well as the periods of sucrose diet increase, but in the same grade, as compared with mitochondria from C rats (Figure 4B).

**Figure 5:**
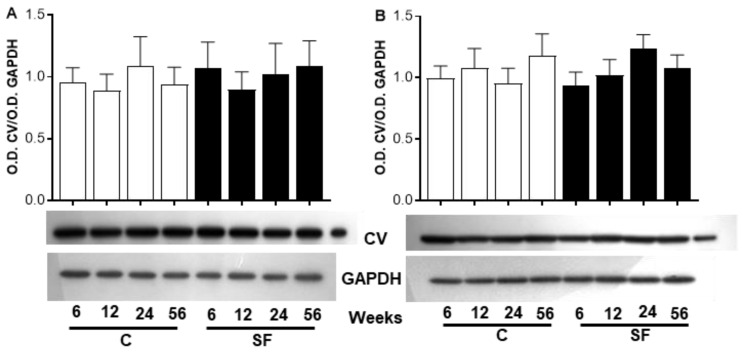
Western blot analysis of mitochondrial ATP synthase 5A (ATP 5A, CV). The open bars correspond to mitochondria from C rats and the black bars correspond to mitochondria from SF rats. Forty µg of proteins from skeletal (**A**) and heart (**B**) muscle homogenates at various periods of sucrose diet and age were electrophoresed through an SDS-PAGE with 15% of acrylamide, electroblotted, and incubated with a cocktail antibody anti-subunits of the respiratory complex comprising the antibody anti-ATP 5A. The Western blot image of the ATP 5A subunit complex shows a protein band and GAPDH in SF compared to C rats. The black bars correspond to SF, and the open bars correspond to C rats. The values correspond to the ratio of the optical density (O.D.) of the subunit to the O.D. of GAPDH. Each value is the mean ± SD of 4 independent homogenate preparations from separate animals.

Figure 5 shows images of the ATP 5A subunits from skeletal muscle at the bottom of panel A and from heart muscle at the bottom of panel B. Figure 5A shows no difference in the ratio of O.D. ATP 5A/O.D. GAPDH between C and SF rats nor between the different periods of sucrose treatment and ages in skeletal muscle.

In the heart muscle, the analysis of the ATP5 subunit did not show a significant difference between the two groups of animals, nor was it affected by the duration of sucrose intake or by aging in CR (Figure 5B).

**Figure 6:**
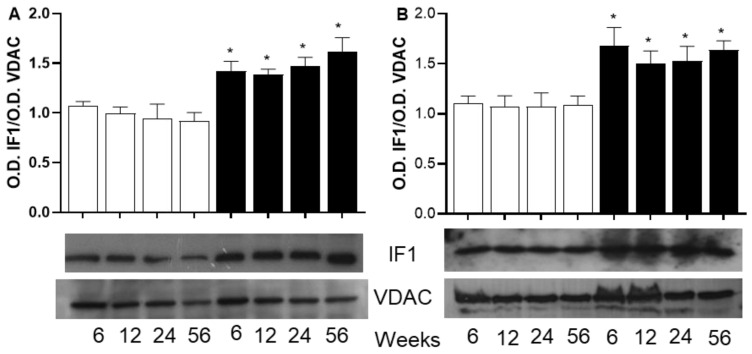
Western blot analysis of ATPase inhibitor factor 1 (IF1) in mitochondria from skeletal muscle (**A**) and from heart muscle (**B**). The open bars correspond to mitochondria from C rats and the black bars correspond to mitochondria from SF rats. Forty micrograms of protein were separated using SDS-PAGE with 12% acrylamide, transferred to a membrane, and treated with anti-IF1 antibody (1/2000). The optical density (OD) of IF1 is normalized to the OD of VDAC as a loading control. The values correspond to the ratio of the OD of the subunit to the OD of VDAC. Each value is the mean ± SD of 4 independent mitochondria preparations from separate animals. * *p* < 0.05 corresponds to the significant difference between C and SF rats at the same period of aging and sucrose treatment.

For the ATPase inhibitory factor 1 (IF1), Figure 6A,B show that the amount of IF1 protein in mitochondria from both skeletal and heart muscle goes up with a sucrose diet, and this increase is not affected by how long the diet lasts.

## 3. Discussion

The increasing statistical differences in the lipid metabolism parameters, such as fat accumulation, plasma FFAs, and TGs, between C and SF rats are associated with the increased duration periods of sucrose treatment. The longer the duration of the sucrose treatment period, the more pronounced the effect on lipid metabolism. The intra-abdominal fat and increased FFA and TG concentrations, at any period of high sucrose consumption, reflect an increased lipogenic activity. In addition, the long and very long periods of sucrose treatment result in an accumulation of palmitic, palmitoleic, and oleic acids in skeletal muscle homogenate more than in heart muscle. Such accumulation of FFA in skeletal muscle is a marker of altered lipid metabolism and lipotoxicity [20], and it is due to the impaired capacity of skeletal muscle to oxidize fatty acids, which contributes to the accumulation of FFA and further impairs fatty acid and glucose metabolism [21]. On the other hand, in cardiac muscle, where ATP synthesis relies more on beta-oxidation of fatty acids, the absence of FFA accumulation is due to the heart’s higher energy demands compared to skeletal muscle. Among the FFAs accumulated in skeletal muscle homogenate are palmitoleic and oleic acids, which are the products of the elongation and desaturation of PA [17]. The elongation and desaturation processes are considered an act of detoxification of PA to favor its incorporation as palmitoleic and oleic acids in TG, which in turn accumulates in adipose and non-adipose tissue to prevent the adverse effects of the excess PA, as reviewed recently [20,22].

**Figure 7:**
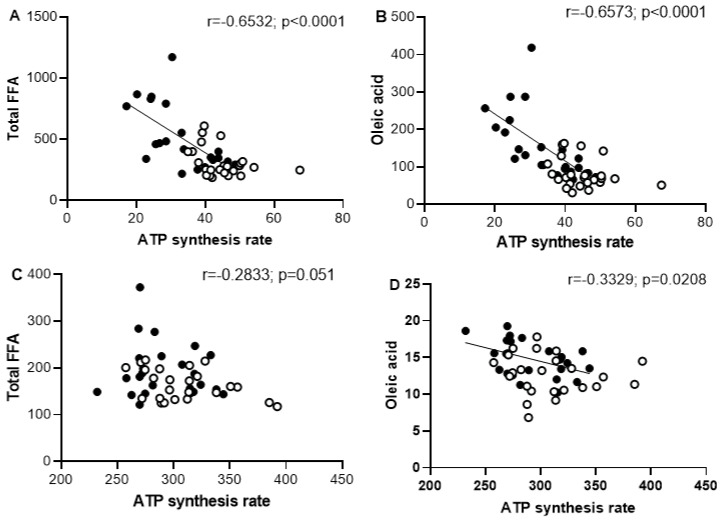
Pearson correlation analysis of total FFA and oleic acid (nmol/mg protein) with ATP synthesis rate (nmol/min/mg protein) in skeletal muscle (**A**,**B**). Additionally, the analysis includes heart muscle (**C**,**D**). Black circles correspond to SF rats, and open circles correspond to C rats.

The increased FFA accumulation in skeletal muscle homogenate, particularly PA and oleic acid after long periods of sucrose intake, is associated with the impaired ATP biosynthesis in mitochondria from skeletal muscle. The negative correlation between skeletal FFA and ATP synthesis rate suggests the influence of FFA on the biosynthesis of ATP in SFR (Figure 7). Unlike skeletal muscle, the heart muscle did not present a significant association between ATP synthesis and FFA accumulation, suggesting a resistance to the sucrose diet induced both alterations.

A smaller buildup of FFA in the heart might be due to the process of beta-oxidation of fatty acids, which is important for producing ATP in the heart. FFAs activated to FA-CoA serve as a substrate source for ATP synthesis after their conversion to acetyl-CoA through mitochondrial β-oxidation [23]. In skeletal muscle, our finding suggests that the reduction in ATP biosynthesis can be due to detrimental effects of FFA on mitochondrial function. FFAs such as palmitic and palmitoleic acids have been described to reduce the mitochondrial membrane potential, affecting the electron transport chain function and mitochondrial ATP synthesis in isolated mitochondria [24]. In addition, mitochondrial dysfunction characterized by uncoupling ATP biosynthesis is well known to be related to fat accumulation and metabolic syndrome [25].

Several mechanisms to decrease ATP synthesis in cells through excessive FFAs have been reported [26]. One of them is the high availability of FFA to activate UCPs, uncoupling OXPHOS and reducing ATP biosynthesis. In our study, the interplay between UCPs and ATP production is demonstrated by using oleic acid and GDP as activators and inhibitors of UCPs, respectively. Oleic acid inhibits ATP biosynthesis in a dose response, and this inhibition is sensitive to GDP, suggesting the participation of UCPs in the uncoupling of the ATP biosynthesis. In addition, mitochondria from both heart and skeletal muscle of SFR exhibit higher ATP biosynthesis inhibition compared to C rats when challenged with oleic acid at low concentration (2.5 µM). This sensitivity to oleic acid increases as well as the duration of sucrose treatment. In both skeletal and heart muscles, UCP2 and UCP3 have been described to influence ATP production and energy expenditure [27]. In mitochondria from heart muscle, our findings indicate that both UCP2 and UCP3 expressions increase with age in mitochondria from C rats equally to those from SF rats and did not change with the period of sucrose treatment as described elsewhere [28]. UCP3 has been described to protect against lipotoxicity by managing fatty acid oxidation and energy dissipation in muscles [7] and improving mitochondrial functionality [6,29]. In heart muscle, the influence of a sucrose-rich diet on the expression of UCP2 and UCP3 involves various molecular mechanisms, including oxidative stress and inflammation, which in turn increase the expression of UCPs as a protective response against mitochondrial lipotoxicity and cell apoptosis [30].

In this study, the higher levels of UCP2 seem to be more connected to age rather than the buildup of fat and free fatty acids, suggesting an adaptive response to mitigate oxidative stress related to aging [31]. In a previous study, a high-sucrose diet for 24 weeks has been described to increase the levels of UCP2 in liver mitochondria in response to enhanced ROS generation [32]. Indeed, the UCP2 overexpression has been described as playing a dual role in obesity. On one hand, UCP2 can protect against oxidative stress by dissipating the proton gradient. On the other hand, its overexpression results in a reduction in ATP biosynthesis [33]. In heart muscle, UCP3 is important for moving non-esterified fatty acid anions out of the mitochondria, which helps stop them from building up and causing harm [34,35]. Moreover, it has been demonstrated that proton leak decreases mitochondrial respiratory efficiency in aged cardiomyocytes [36] and impacts the ATP synthesis rate, which may contribute to heart dysfunction in aging. On the other hand, it has been reported that in muscle cells, UCP3 and 2 overexpression do not influence the ATP biosynthesis nor the mitochondria function [37], but they do promote fatty acid oxidation, especially under conditions of high fatty acid availability, thereby preventing cytosolic triglyceride storage [6].

In skeletal muscle form SF rats, FFA accumulation may contribute to energy regulation by uncoupling OXPHOS or by the direct inhibition of adenine nucleotide translocase (ANT) and ATP synthesis, thereby modulating ATP production [38]. Our findings show that the reduction in ATP synthesis in skeletal muscle correlates inversely with the accumulation of total FFA and oleic acid (Figure 7). When total FFA or oleic acid levels reached the maximal concentrations per mg protein, ATP biosynthesis was significantly depressed. This means that, as the amount of fatty acid increases, its ability to regulate uncoupling or inhibit ATP biosynthesis also increases.

In skeletal muscle of SF rats, the decrease in ATP biosynthesis in mitochondria can also be due to the possible alterations in the content or to the changes in mitochondrial respiratory chain complexes, which need further investigation. Mitochondrial ATP5A, a marker of complex V and a key step in ATP generation, is carried out by a set of protein complexes in the electron transport chain, which were analyzed in the current study. The lack of differences between mitochondria from C and SF rats in the ATP5 (complex V) suggests that the decreased ATP synthesis is not related to the amount of ATP5 subunit, which reflects the expression of ATP synthase.

Mitochondrial ATP biosynthesis can also be regulated by the ATP synthase inhibitory factor 1 (IF1), a protein found to be increased in the heart and skeletal muscle of SF rats. The IF1 is a nuclear-encoded protein that interacts with and suppresses the hydrolysis activity of ATP synthase. When ATP synthase works in reverse to break down ATP, especially when free fatty acids (FFAs) disrupt the inner mitochondrial membrane, IF1 may help protect the cell by stopping ATP synthase and preventing the loss of ATP and cell death. This situation often happens in cells or mitochondria that are exposed to uncouplers, similar to low oxygen conditions like cardiac ischemia, where a lack of oxygen causes a drop in membrane potential [39].

In obesity-related metabolic disorders, the role of IF1 is not well understood and needs further investigation. However, the higher levels of IF1 in the mitochondria from both tissues of SF rats suggest that IF1 might help preserve ATP levels, which could drop significantly due to uncoupling of the mitochondria and blocking OXPHOS. These observations suggest that IF1 may play a regulatory role in energy metabolism, which could influence diseases such as metabolic syndrome and requires further study.

In summary, the long period of sucrose-based diet altered ATP biosynthesis in skeletal muscle mitochondria, which is related to the accumulation of free fatty acids caused by prolonged periods of unhealthy diet. As the amount of FFA increases, they further disrupt ATP production. In addition, the sensitivity of ATP biosynthesis to oleic acid as an FFA increases with the duration of sucrose treatment. In skeletal and cardiac muscles, this sensitivity is attributed to the expression of UCP2 and UCP3 proteins, which have been described as influencing ATP production and energy expenditure. This study also established that the increase in IF1 expression in all groups receiving a sucrose-rich diet is independent of the duration of sucrose treatment.

## 4. Experimental Design

### 4.1. Animals and Their Treatment

The experiments followed the rules set by the Mexican Federal Regulation for Animal Experimentation and Care (NOM-062-ZOO-2001) and had permission from the Committee for Care and Use of Laboratory Animals of the Nacional Institute of Cardiology Ignacio Chavez (CICUAL) under the number INC/CICUAL/003/2023. Male Wistar rats, aged 21 days and weighing 65 ± 5 g, were used for this study. The animals were randomly assigned to one of two experimental groups: The sucrose-fed (SF) rats received 30% commercially refined sugar dissolved in their drinking water ad libitum for different periods of 6, 12, 24, and 56 weeks. The control rats (C) were administered only tap water. All animals had ad libitum access to commercial rat chow (PMI Nutrition International, Inc., LabDiet 5008, Richmond, IN, USA) and were housed in a controlled environment maintained at 22–24 °C with a 12 h light–dark cycle.

Following the overnight fasting period, the animals were weighed and subsequently euthanized by decapitation; the heart, soleus, and red gastrocnemius muscles were promptly excised and placed in ice-cold isolation buffer. Intra-abdominal fat was meticulously dissected from the retroperitoneal cavity and immediately weighed. It is noteworthy that visceral and duodenal fat was excluded from this dissection procedure.

### 4.2. Measurement of Plasma Glucose, Cholesterol, and Triglycerides

The venous blood was promptly drawn into a tube containing EDTA (0.1%) and immediately subjected to centrifugation at 600× *g* for a duration of 20 min at a temperature of 4 °C. The resultant plasma was subsequently separated and preserved at −70 °C until the analyses of FFA, triglycerides, cholesterol, and glucose were conducted. The amounts of plasma triglycerides, cholesterol, and glucose were measured using special color tests with an immunoturbidimetry method on a Hitachi analyzer (Hitachi Ltd., Tokyo, Japan) [40].

### 4.3. FFA Extraction and Determination by Gas Chromatography (GC)

FFAs were extracted from 100 µL of plasma and from 10 mg of protein of tissue homogenates in the presence of 50 and 20 µg of heptadecanoic acid as an internal standard, respectively. The extraction was performed using chloroform-methanol (2:1 *v/v*) containing 0.002% BHT as described by Folch et al. [41]. The obtained lipid residue was dissolved in 1 mL methanol containing 0.1 mL 2,2-dimethoxypropane and 0.01 mL concentrated HSO_4_ to transform FFA to their corresponding methyl esters at room temperature for 15 min according to the method of Tserng et al. [42] modified in our laboratory [43]. The concentration and composition of FFA methyl esters were evaluated by GC as described elsewhere [43].

### 4.4. Mitochondria Preparation

Mitochondria were isolated from heart and skeletal muscle by differential centrifugation. Tissues were quickly removed and placed in a cold solution containing 250 mM sucrose, 10 mM of HEPES, 1 mM of EGTA (pH: 7.4), 1 mg/mL nagarse, or 1 mg/mL trypsin to break heart and skeletal muscle tissues, respectively, and to release mitochondria, which then are separated using differential centrifugation [44]. The final mitochondrial pellet was suspended in cold buffer containing 250 mM sucrose and 10 mM HEPES (pH: 7.4). Sample protein content was quantified by Bradford’s method [45], using bovine serum albumin for the standard curve. The fresh mitochondria were used for the following assays of ATP biosynthesis.

### 4.5. Mitochondrial Oxygen Consumption Rate (OCR)

Mitochondrial OCR was measured by using a Clark-type O_2_ electrode and by incubating 0.25 mg protein/mL of fresh mitochondria in an air-saturated medium containing 125 mM KCl, 10 mM HEPES (pH adjusted to 7.4 with KOH), 10 mM EGTA, 2 mM K_2_HPO_4_, and 5 mM MgCl. Oxygen consumption rate was assessed by energizing mitochondria with succinate (10 mM) at 30 °C. ADP (250 µM) was added to evaluate state III. State IV corresponds to oxygen uptake after ADP exhaustion.

### 4.6. ATP Biosynthesis

ATP synthesis rate was measured as follows: 0.25 mg of skeletal muscle and 0.3 mg of protein in the case of the heart were incubated in 20 mM Tris-HCl, 0.15 M sucrose, 1 mM ADP, 20 mM Pi, 5 mM MgCl_2_, 100 µM diadenosinepentaphosphate to inhibit adenylate kinase, 10 mM glucose, and 37.5 units/mL hexokinase type IV (Sigma Aldrich, Mexico City, Mexico), pH 7.5. Samples (0.4 mL) were incubated at 37 °C with vigorous shaking for maximal oxygenation. The reaction was started with 20 mM succinate and arrested at the desired time with 25 mM EDTA and 10 µM CCCP and immediately transferred to ice. Samples were transferred to Eppendorf tubes, boiled for 10 min, and centrifuged (10,000× *g*, 10 min) to remove denatured protein. In supernatants, the synthesized glucose-6-phosphate was oxidized by NADP in the presence of three units of glucose-6-phosphate dehydrogenase. NADPH formation was monitored at 340 nm until completion. ATP formation was calculated after correction for trapping efficiency of hexokinase, which was determined by adding known amounts of ATP to the reaction mixture in the absence of succinate [46]. In other experiments in the series, the mitochondria were incubated with different concentrations of oleic acid (2.5, 5, and 10 µM), and in the presence of 0.5 mM GDP, an inhibitor of UCPs was added to the reaction medium at the start of the experiment.

### 4.7. Western Blot Analysis of UCPs, IF1, and Mitochondrial Respiratory Complex V

Forty μg of mitochondria or tissue homogenate protein was loaded in SDS-PAGE gel whose acrylamide percentage depends on the molecular weight of proteins to analyze, as indicated in figure legends. The electrophoresis was run for 3 h at 120 V. The protein transfer was performed on a PVDF membrane (polyvinylidene fluoride, pore size 0.22 µM, Millipore Corporation, Auburn, WA, USA) at 20 V for 30 min in a wet chamber of transfer (Invitrogen, Trans Blot SD, Waltham, MA, USA). The non-specific protein detection was reduced by blocking membranes in tris-buffered saline (TBS) (25 mM tris base, 150 mM NaCl) solution containing 1% albumin-free fatty acid and 0.1% Tween 20 for one hour at room temperature. After washing with TBS three times, the membranes were incubated with the total OXPHOS rodent WB Antibody Cocktail (Abcam) at a concentration of 1:3000. This cocktail contains antibodies against complex V ATP 5A subunit (ATP synthase 5A). For ATPase inhibitor protein F1 (IF1), UCP2, and UCP3, the antibodies against IF1 and against UCP2 and UCP3 were also purchased from ABCAM Corporation and were used at a dilution of 1:1000 in TBS–Tween (0.1%) under gentle agitation overnight at 4 °C. At the end of incubation, the membranes were washed three times with TBS–Tween (0.1%) for 5 min each and twice with TBS for 10 min each. As a secondary antibody, anti-rabbit IgG peroxidase conjugate was used at a dilution of 1:1000 in TBS–Tween (0.1%) at room temperature for 2 h. The membranes were subsequently washed 5 times, twice with TBS–Tween (0.1%) and three times with only TBS; proteins were revealed by chemiluminescent reagent (Immobilon, Millipore, Burlington, MA, USA). Membranes were exposed to image plates (BioMax Kodak, Carestream Health, Rochester, NY, USA) for 5 min. The bands obtained were analyzed by a UVP image analyzer (UVP Inc., Upland, CA, USA). For loading control, the same membrane was incubated in a solution of 40 mM SDS and 250 mM glycine (pH: 2) for 30 min to remove reagents and antibodies used above. Subsequently incubated in TBS–Tween (0.1%) solution containing 1% free fatty acid albumin for 1 h, the membrane was then treated as described above, but in this case, it was treated with VDAC and GAPDH antibodies as a control load according to the tissue analyzed.

### 4.8. Statistical Analysis

All statistical analyses were performed using GraphPad Prism version 8.2.1 for Windows (GraphPad Software, Boston, MA, USA). All values are presented as means ± SD. The differences between groups were determined by one-way ANOVA for selected variables followed by an ad hoc Tukey test. The number of animals used for each analysis is indicated in the figure and table legends. The differences between group means were considered significant when *p* < 0.05.

## 5. Conclusions

This study demonstrates that a sucrose-rich diet increases FFA concentration in skeletal and cardiac muscles. It also shows that these FFAs are inversely correlated with ATP synthesis. Furthermore, it has been demonstrated that the effect of FFA on ATP synthesis involves mitochondrial UCP2 and UCP3. The expression of UCPs is considered a response to stress factors, such as increased FFA and ROS, which are characteristics of mitochondrial stress that can also affect ATP synthesis. These results suggest that the two processes of increased FFA from lipogenesis and mitochondrial stress are closely related. Although this is a new finding regarding IF1 protein levels, the decrease in ATP synthesis may be multifactorial, involving increased fatty acid synthesis and mitochondrial stress. Further research is needed to delineate these pathways and their respective contributions to ATP synthesis in the context of a sucrose-rich diet.

## Figures and Tables

**Table 1:** General characteristics of animals with and without sucrose diet intake during different periods.

Animals		C				SF			
	Weeks	6	12	24	56	6	12	24	56
Variables									
Body mass (g)		284.8 ± 20.1	384.15 ± 2.7	552 ± 33.5	575.7 ± 46.4	259.1 ± 17.7	388 ± 35.3	572.9 ± 37.4 ^$^	716.8 ± 50.3 *^$^
Intrabdominal fat (g)		2.3 ± 0.9	4.3 ± 0.79	5.9 ± 1.2	8.6 ± 2.9	3.4 ± 0.7	8.6 ± 1.5 *^$^	14.9 ± 2.6 *^$^	25.6 ± 5.96 *^$^
Triglycerides (mM)		0.8 ± 0.1	0.8 ± 0.1	0.9 ± 0.2	1.2 ± 0.2	1.2 ± 0.1 *	1.4 ± 0.2 *	1.8 ± 0.3 *^$^	1.7 ± 0.2 *^$^
Total FFA (µM)		243.2 ± 40.8	248.9 ± 38.9	312.8 ± 106.1	479.6 ± 113.2	353.7 ± 41.7	319.7 ± 74.4	461.1 ± 87.7 *^$^	830.2 ± 202.3 *^$^
Glucose (mM)		6.2 ± 0.8	4.8 ± 1.9	4.9 ± 2.1	5.8 ± 0.7	5.5 ± 1.1	4.6 ± 1.8	4.9 ± 1.9	6.2 ± 0.7
Cholesterol (mM)		1.3 ± 0.2	1.5 ± 0.1	1.4 ± 0.2	1.6 ± 0.2	1.7 ± 0.2	1.4 ± 0.2	1.2 ± 0.2	1.6 ± 0.3

All values correspond to mean ± SD of 7 to 8 animals. C corresponds to control rats, and SF corresponds to sucrose-fed rats. The differences between groups were determined by one-way ANOVA for selected variables followed by the Tukey test. * *p* < 0.01 corresponds to C vs. SF at the same age or period of sucrose treatment, and ^$^ *p* < 0.01 between ages and between periods of sucrose treatment in the same group. The differences between C and SF rats increase as well as the periods of sucrose treatment increase.

**Table 2:** Mitochondria oxygen consumption rate (OCR) oxidizing succinate.

Animals		C				SF			
	Weeks	6	12	24	56	6	12	24	56
OCR									
Mitochondria from skeletal muscle									
State III		121.0 ± 7.2	108.2 ± 16.0 *	79.2 ± 7.1 *	69.9 ± 10.8 **	115.6 ± 9.15	101.1 ± 13.6	70.9 ± 13.0 ^$^	50.08 ± 7.1 ^$$^
State IV		22.6 ± 5.3	23.1 ± 6.5	20.8 ± 2.8	21.4 ± 3.2	22.1 ± 3.1	22.1 ± 4.4	26.1 ± 2.3 ^&^	27.4 ± 2.8 ^&&^
Mitochondria from heart muscle									
State III		118.8 ± 10.2	98.6 ± 9.2	89.5 ± 24.8 *	61.7 ± 8.9 **	95.6 ± 14.8	92.1 ± 18.2	60.4 ± 9.7 ^$^	50.8 ± 16.4 ^$$^
State IV		22.4 ± 3.3	21.7 ± 2.8	18.5 ± 3.4	19.3 ± 3.8	21.4 ± 4.6	22.8 ± 3.5 ^&^	21.3 ± 2.4 ^&^	23.0 ± 6.6 ^&^

All values correspond to mean ± SD of 7 to 8 animals. OCR is expressed as nmolO_2_/min/mg protein. C corresponds to control rats, and SF corresponds to sucrose-fed rats. The differences between groups were determined by one-way ANOVA for selected variables followed by the Tukey ad hoc test. * *p* < 0.01 corresponds to C at 24 weeka vs C ata 6 weeks. ** *p* < 0.01 corresponds to C 56 weeks vs. C at 6 weeks. ^&^ *p* < 0.05 corresponds to the difference between SF and C at 24 weeks. ^&&^ *p* < 0.01 correspond to the difference between SF at 56 and 6 weeks. ^$^ *p* < 0.01 corresponds to the difference between SF at 24 weeks and 6 weeks. ^$$^ *p* < 0.05 corresponds to the difference between SF at 56 and 6 weeks of sucrose treatment.

## Data Availability

No new data were created.
